# Non-parental caregivers, low maternal education, gastrointestinal problems and high blood lead level: predictors related to the severity of autism spectrum disorder in Northeast China

**DOI:** 10.1186/s12887-021-03086-0

**Published:** 2022-01-03

**Authors:** Han-Yu Dong, Jun-Yan Feng, Hong-Hua Li, Xiao-Jing Yue, Fei-Yong Jia

**Affiliations:** Department of Developmental and Behavioral Pediatrics, the First Hospital of Jilin University, Changchun, 130021 Jilin Province China

**Keywords:** Autism, Environment, Severity, Socioeconomic, Nutritional, Lead

## Abstract

**Background:**

The prevalence of autism spectrum disorder (ASD) has increased rapidly in recent years. Environmental factors may play an important role in the pathogenesis of ASD. These factors may include socioeconomic factors, nutritional factors, heavy metal exposure, air pollution, etc. Our aim is to analyze possible environmental factors associated with the severity of ASD.

**Methods:**

All participating children were divided into two groups (mild and moderate/severe) according to the severity of their symptoms, as determined by their Childhood Autism Rating Scale (CARS) scores. The socioeconomic, demographic factors and the nutritional factors that may affect the severity of ASD were included in the logistic regression to analyze whether they were predictors that affected the severity of ASD.

**Results:**

Logistic regression showed that caregivers(*P* = 0.042), maternal education (*P* = 0.030), gastrointestinal problems (*P* = 0.041) and a high serum concentration of lead (*P* = 0.003) were statistically significantly associated with ASD severity.

**Conclusion:**

Many environmental factors affect the severity of ASD. We concluded that non-parental caregivers, low maternal education, gastrointestinal problems and high blood lead level maybe predictors that affected the severity of ASD in northeast China.

## Background

Autism spectrum disorder (ASD) was originally defined by Leo Kanner in 1943 and is characterized by persistent deficits in social communication and interaction and stereotyped or repetitive patterns of behavior, interests or activities. ASD is classified as a neurodevelopmental disorder in the Diagnostic and Statistical Manual of Mental Disorders 5th Edition (DSM-5). The prevalence of ASD is increasing so rapidly that it has led to the development of many social issues and placed a heavy burden on families.

Currently, the cause of ASD remains unclear. In recent years, the prevalence of ASD has increased significantly. This increase is partly due to changes in diagnostic criteria, reductions in the misdiagnosis rate, and increases in the consultation rate, but the reasons for the increase are not limited to these factors. In addition, large-scale mutations that generate ASD-related pathogenic genes in a short time are obviously unlikely, and we have reason to believe that environmental factors may play an important role in the pathogenesis of ASD. So it is suggested that ASD is the consequence of both genetic factors and environmental factors. These factors may include nutritional factors, heavy metal exposure, air pollution, socioeconomic factors, etc. [1].

Nutritional factors include omega-3 fatty acids [2–4], vitamin A [5, 6], vitamin D [7, 8], folic acid [9, 10], iron [11, 12] and other micronutrients (like Zn and Cu [13–15]). Abnormal metabolism may be linked to neurological and behavioral disorders and ASD [16], providing ideas for the nutritional treatment of ASD. In recent years, problems related to environmental toxicants have become increasingly prominent. Heavy metals, especially lead and mercury, are among the environmental toxicant predictors for ASD [17–19]. Socioeconomic factors include parental education [20, 21], family income [22], caregivers, birth order [23], siblings [24], etc. An understanding of socioeconomic factors can be very helpful for the management of autism and for social work, including social welfare practices.

In China, some studies have focused on the influence of various environmental factors on the severity of autism symptoms. Liu [25] conducted a case-control study with 81 children with ASD and showed that maternal occupational toxicant exposure, diseases during pregnancy, and living in an impoverished area the time of birth may be specific predictors associated with ASD. Shen [26] collected a basic medical history and information regarding maternal prepregnancy and pregnancy conditions and reported that maternal prepregnancy BMI might not be associated with autism risk. Zhang [27] conducted a case-control study of 190 Han children and showed that 9 prenatal and perinatal predictors were associated with ASD. Our team’s previous research also found that vitamin D levels had some relationship with ASD symptoms [28].

However, there is still a lack of comprehensive analysis of the influence of socioeconomic factors, nutritional factors, and heavy metal elements on the severity of ASD symptoms in a large sample of children with ASD in northeast China. These factors are clearly related to race, region, and social environment. Therefore, regional research may be more meaningful for understanding the role of environmental predictors for ASD in the pathological processes of ASD in our own region. For these reasons, we conducted this retrospective study to identify possible environmental predictors related to the severity of ASD.

## Methods

### Participants

We retrospectively analyzed 512 children with ASD (417 boys and 95 girls) ranging in age from 2 to 13 years [median (Q1-Q3), 3.3(2.7,4.3)]. All the children were diagnosed according to the DSM-5 criteria for the first time and were confirmed to not have fragile X syndrome, Rett syndrome or other moderate/severe neurological diseases, such as epilepsy, by developmental and behavioral pediatricians in the First Hospital of Jilin University from October 2017 to January 2020. All the children were divided into two groups according to the severity of their symptoms, as determined by their Childhood Autism Rating Scale (CARS) scores. The CARS consists of 15 scales, and each scale is scored on a continuum from normal to severely abnormal. The CARS was developed by Schopler and Reichler and is used as a diagnostic scale. CARS scores range from 15 to 60 points, and higher scores in this scale indicate worse ASD symptoms.

The average CARS score of the children with ASD was 30.9 ± 4.8 (22–47) points. Children with CARS scores below 30.9 were included in the mild group, and children with CARS scores greater than 30.9 were included in the moderate/severe group. The mild group included 249 children (208 boys and 41 girls), and the moderate/severe group included 263 children (209 boys and 54 girls). The mean CARS scores of these groups were 27.0 ± 2.78 (22–30.5) and 34.6 ± 3.1 (33–47), respectively. The ethics committee of our hospital approved this research program.

### Evaluations and measurements

All children in this study were assessed via socioeconomic and demographic profile surveys, symptom evaluation scales and blood tests.

The socioeconomic and demographic information that was collected included name, sex (male or female), age, birth date (year, month and day), place of residence (urban or rural area), caregivers (parents, grandparents or both), siblings, age of parents during pregnancy, education level of parents, household income, family history of mental illness, vitamin intake during pregnancy (none, folic acid or multivitamins), mode of delivery (eutocia or cesarean), presence of eating problems, presence of sleeping problems, presence of gastrointestinal problems, and comorbidity with attention deficit hyperactivity disorder (ADHD).

The symptom evaluation scales included the Autism Behavior Checklist (ABC), the CARS, and the Autism Treatment Evaluation Checklist (ATEC). The CARS scale has been described above. The ABC is a 57-item screening checklist for autism containing 5 subscales (body behavior, sensory, self-care, language and social interaction). The ATEC was designed to measure treatment effects and has four subscales: speech/language communication, sociability, sensory/cognitive awareness and health/physical/behavior; the ATEC is usually used to evaluate treatment effects in children with ASD. The reliability and validity of the ABC, CARS and ATEC are sufficiently good, reflecting the scales’ usefulness for clinical diagnosis and the evaluation of ASD symptoms [29]. The survey was conducted by doctors and families together, including children and their parents. The ABC and ATEC scales are designed to be administered via parent interviews. The CARS requires the observation of children with ASD in a consulting room. Higher scores in these scales indicate worse ASD symptoms.

Blood tests included measurements of vitamins A, D, and E; copper; zinc; iron; and lead. The serum concentrations of vitamins A, D and E were detected by high-performance liquid chromatography (HPLC). The serum concentrations of copper, zinc, iron, and lead were detected by graphite furnace atomic absorption spectrometry (AAS). All samples were tested by Guangzhou KingMed Diagnostics Group Co., Ltd. (KingMed Diagnostics, SSE 603882).

### Statistical analysis

We used the Statistical Package for the Social Sciences (SPSS) 19.0 (SPSS for Windows, SPSS Inc. Chicago) to analyze the data.

We compared the socioeconomic, demographic profiles and blood tests of the mild group and moderate/severe group to determine whether there were factors that differed between the two groups. Continuous variables with normal distributions are represented as means ± standard deviations (SDs) and were compared by Student’s t-test. Continuous variables with nonnormal distributions are represented as medians (Q1-Q3) and were compared using Wilcoxon’s rank-sum test. Categorical variables are represented as frequencies (percentages) and were compared using the χ2 test.

The socioeconomic, demographic and nutritional factors that had statistic differences between the two groups were included in the logistic regression to analyze whether these factors affected the severity of ASD. The regression analysis is versus the CARS score.

## Results

Table 1 shows the comparison of the socioeconomic and demographic profiles of the mild group and the moderate/severe group. The mild group consisted of 208 boys (83.5%) and 41 girls (16.5%), and the moderate/severe group consisted of 209 boys (79.5%) and 54 girls (20.5%). A statistically significant difference was found for the following 6 factors: age (*P* = 0.01), place of residence (*P* = 0.034), caregivers (*P* = 0.046), maternal and paternal education (*P* = 0.034 and 0.008, respectively) and gastrointestinal problems (*P* = 0.03). The children in the mild group were older than those in the moderate/severe group. Regarding place of residence, more families in the mild group lived in urban areas; the parents of children in the mild group also had a higher degree of education than the parents of children in the moderate/severe group. The children in the mild group were more likely to have parents as their main caregivers and had a lower rate of gastrointestinal problems than those in the moderate/severe group.

**Table 1:** Comparison of the socioeconomic and demographic profiles of the mild and moderate/severe groups

Variables	Mild group N (%)		Moderate/severe group N (%)	Z/χ^**2**^	***P***
**Sex**				1.400	0.237
Male	208 (83.5%)		209 (79.5%)		
Female	41 (16.5%)		54 (20.5%)		
**Age (**median (Q1-Q3)**)**	3.5 (3–5)		3 (2.5–4)	−3.250	0.010*
**Birth month**				2.961	0.398
Nov-Jan	66 (26.5%)		79 (30.0%)		
Feb-Apr	51 (20.5%)		50 (19.0%)		
May-Jul	65 (26.1%)		54 (20.5%)		
Aug-Oct	67 (26.9%)		80 (30.4%)		
**Place of residence**				4.505	0.034*
Urban	193 (77.5%)		182 (69.2%)		
Rural	56 (22.5%)		81 (30.8%)		
**Caregivers**				7.986	0.046*
Parents	159 (63.9%)		137 (52.1%)		
Grandparents	66 (26.5%)		93 (35.4%)		
Both parents and grandparents	20 (8.0%)		30 (11.4%)		
Others	4 (1.6%)		3 (1.1%)		
**Siblings**				1.762	0.184
Yes	52 (20.9%)		68 (25.9%)		
No	197 (79.1%)		195 (74.1%)		
**Age of mother during pregnancy** (median (Q1-Q3))	28 (25–30)		28 (24–31.75)	−0.710	0.478
**Age of father during pregnancy** (median (Q1-Q3))	29.5 (27–33)		29 (26–33)	−0.297	0.767
**Maternal education**				7.132	0.008*
Junior college or above	109 (43.8%)		85 (32.3%)		
Senior high school or below	140 (56.2%)		178 (67.7%)		
**Paternal education**				4.506	0.034*
Junior college or above	104 (41.8%)		86 (32.7%)		
Senior high school or below	145 (58.2%)		177 (67.3)		
**Household income (10,000 yuan)** (median (Q1-Q3))	5 (4–7)		5 (4–6)	−1.918	0.055
**Family history of mental illness**				0.001	0.973
Yes	31 (12.4%)		33 (12.5%)		
No	218 (87.6%)		230 (87.5%)		
**Vitamin intake during pregnancy**				2.795	0.424
None	29 (11.6%)		43 (16.3%)		
Folic acid	139 (55.8%)		139 (52.9%)		
Multivitamins	57 (22.9%)		53 (20.2%)		
Others	24 (9.6%)		28 (10.6%)		
**Mode of delivery**				0.026	0.872
Vaginal	85 (34.1%)		88 (33.5%)		
Cesarean	164 (65.9%)		175 (66.5%)		
**Eating problems**				0.247	0.619
Yes	111 (44.6%)		123 (46.8%)		
No	138 (55.4%)		140 (53.2%)		
**Sleeping problems**				0.529	0.467
Yes	74 (29.7%)		86 (32.7%)		
No	175 (70.3%)		177 (67.3%)		
**Gastrointestinal problems**				4.709	0.030*
Yes	53 (21.3%)		78 (29.7%)		
No	196 (78.7%)		185 (70.3%)		
**Comorbidity with ADHD**				0.250	0.617
Yes	30 (12.0%)		28 (10.6%)		
No	219 (88.0%)		235 (89.4%)		

*P<0.05

Table 2 shows the comparison of the blood tests between the two groups in the mild and moderate/severe group. It indicates that the difference in serum concentration of lead between the two groups is statistically significant(t = − 3.489, *P* = 0.001).

**Table 2:** Comparison of the blood tests of the mild and moderate/severe groups

Blood tests	Mild group (mean ± SD)	Moderate/severe group (mean ± SD)	t	***P***
**Vitamin A (mg/L)**	**0.35 ± 0.08**	**0.49 ± 2.17**	**0.105**	**0.917**
**Vitamin D (ng/mL)**	**26.05 ± 8.79**	**26.85 ± 9.25**	**−1.119**	**0.264**
**Vitamin E (mg/L)**	**7.94 ± 2.79**	**7.96 ± 2.60**	**−0.347**	**0.729**
**Cu (μg/dl)**	**116.52 ± 26.94**	**117.28 ± 26.68**	**−0.114**	**0.909**
**Zn (mg/L)**	**4.63 ± 0.78**	**4.55 ± 0.75**	**0.932**	**0.352**
**Fe (mg/L)**	**442.22 ± 42.88**	**464.54 ± 40.20**	**−0.930**	**0.353**
**Pb (μg/dl)**	**2.58 ± 1.08**	**3.25 ± 1.89**	**−3.489**	0.001*

*P<0.05

Therefore, we entered age, place of residence, caregivers, parental education level, gastrointestinal problems, and lead into the logistic regression model to determine whether these covariates have an independent effect on the outcome. The categorical variables were coded as follows: place of residence (Urban = 0, Rural = 1), caregivers (Grandparents and others = 0, Parents = 1), parental education level (Junior college or above = 0, Senior high school or below = 1), and gastrointestinal problems (No = 0, Yes = 1). The results show that caregivers(*P* = 0.042), maternal education (*P* = 0.030), gastrointestinal problems (*P* = 0.041) and a high serum concentration of lead (*P* = 0.003) are statistically significant, indicating that non-parental caregivers, low maternal education, the presence of gastrointestinal problems and a high serum concentration of lead are predictors related to the severity of ASD symptoms (see detail in Table 3). The R2 of this logistic regression model is 0.273.

**Table 3:** The results of logistic regression

	B	Wals	***P***
**Age**	**−0.079**	**1.234**	**0.267**
**Place of residence**	**0.546**	**2.476**	**0.116**
**Caregivers**	**−0.509**	**0.262**	0.042*
**Maternal education**	**0.617**	**2.879**	0.030*
**Paternal education**	**0.161**	**0.199**	**0.656**
**Gastrointestinal problems**	**0.616**	**4.189**	0.041*
**Pb**	**0.033**	**8.662**	0.003*

*P<0.05

## Discussion

ASD onset occurs in early infancy. ASD is a chronic neurodevelopmental disorder and is now regarded as the consequence of both genetic and environmental factors. In past decades, evidence from twin sibling studies has shown that ASD has a strong inherited tendency [30–32]. Unfortunately, the relationship between genotype and phenotype is not as clear, and copy number variation may be associated with not only ASD but with many other kinds of mental disorders, such as ADHD or schizophrenia. Some disorders are associated with epigenetics. Epigenetics is the result of the interaction between a specific genotype and the environment. It indicates the importance of the environment. In recent years, environmental factors have been viewed as increasingly important in the pathogenesis of ASD.

Our results showed that non-parental caregivers, low maternal education, gastrointestinal problems and high blood lead levels are predictors related to the severity of autism spectrum disorder in northeast China.

### Caregivers

Several decades ago, there was a popular theory called the “refrigerator mother” theory, which suggested that ASD was caused by mothers’ behavior. This theory has been overturned, and we now know that both genetic factors and environmental factors play important roles. However, caregivers, as an aspect of environmental factors, may also affect the severity of ASD. Hobson’s study showed that the severity of ASD was correlated with the quality of parent-child interaction [33]. Autism severity may interact with features of the caregiver-child interaction; it was shown that children with autism were less likely than children without autism to smile in response to their caregivers’ smiles and that their caregivers smiled at them less frequently and were less likely to smile in response to their children’s smiles than caregivers of children without autism [34]. In China, the situation is slightly different. People experience heavy work pressure to provide family financial resources, so grandparents are the main caregivers of children in some families. However, grandparents focus more on daily life activities such as eating and sleeping and pay less attention to children’s emotion, socialization and behavioral development. In addition, grandparents are so fond of their grandchildren that they often cannot make objective appraisals about deficiencies in children’s ability. Consistent with these observations, the mild symptom group had a higher proportion of parents as their main caregivers and a lower proportion of grandparents as their main caregivers. However, a limitation of this study was that we did not evaluate caregiver-child interactions in detail but analyzed only the possible reasons for the proportions of caregivers in each group.

### Maternal education

Higher maternal and paternal education levels were was associated with milder symptoms. Parents with higher education levels can pay more attention to prenatal care, provide a good family environment, and adopt a reasonable parenting style. These factors can offset the poor performance of children with serious genetic susceptibility to ASD, and parents with higher education levels can recognize earlier that their children are not typically developing. Mandell et al. reported that a high parental education level is a protective factor against ASD [35]. In our study, there were differences in maternal and paternal education between the two groups, but paternal education level was not statistically significant in the logistic regression model. This suggests that there was a stronger correlation between maternal education and autism severity. It was because in most families, the education and rearing of young children is mainly the responsibility of women, whereas men are more often responsible for external issues, such as maintaining the family’s economic status [36].

### Gastrointestinal problems

Gastrointestinal symptoms were strongly correlated with ASD symptom severity. Children with severe symptoms are likely to have a much higher proportion of accompanying gastrointestinal problems [37]. These gastrointestinal problems may be the result of different intestinal bacteria and may mediate inflammation and immunological processes that affect the brain [38]. It is also possible that the stereotyped eating behavior of children with ASD has caused gastrointestinal problems in children with ASD. The causal relationship between ASD and gastrointestinal problems cannot be clarified in this study. There are various gastrointestinal symptoms, including constipation, diarrhea, foul-smelling stool, flatulence and abdominal pain. However, we simply analyzed whether the children had gastrointestinal problems and did not perform a detailed evaluation of the kinds and severity of symptoms. In addition, we did not conduct a detailed analysis of gastrointestinal probiotics, immune- and inflammatory-related indicators or the mechanisms related to their relationships with the nervous system. These factors may constitute a future research direction. For human mental health, an important and popular dictum is “fix your gut, fix your brain.” [39].

### Lead

Toxic heavy metals, such as lead and mercury, may affect the developing brain. Lead has been identified as a main neurotoxicant environmental trigger for ASD because it induces neuroinflammation and autoimmunity [40]. Afaf reported that significantly elevated mercury and lead levels were found in the red blood cells of patients with ASD compared with healthy controls [41]. However, related findings have been inconsistent, and Li reported that higher levels of only mercury and arsenic were observed in children with ASD and that their lead levels were not different [42]. The research of Rahbar showed a higher geometric mean blood lead concentration for typically developing controls than for individuals with ASD (2.73 μg/dL vs. 2.25 μg/dL; *p* < 0.05, [43]. Furthermore, studies have found that the severity of autism is also related to an increase in urinary porphyrins (a biological marker related to lead toxicity) [44]. Our results indicated a relationship between blood lead concentration and ASD symptoms, and lead level was included in the final equation, which illustrated that lead level may an environmental factor that affected the severity of ASD. The latest review results [45] show that blood lead levels in children with ASD are higher than those in the control group. Studies [46] have shown that the urine lead of ASD children is lower than that of normal children, indicating that ASD children have poor lead excretion ability, which causes the accumulation of lead in the body, which may cause neurotoxicity. However, our study did not examine the soil, water and industrial production in this region that may affect the level of lead in the environment, which is one of the limitations of this study and needs to be further explored.

### Limitations

Our study had several limitations.

First, we did not recruit typically developing children as a control group. Instead, we reviewed the literature, studied environmental factors that might be predictors for autism and analyzed which factors were actually associated with the severity of autism symptoms.

Second, we used the CARS to evaluate the severity of ASD symptoms, but the CARS is not a structural scale and is somewhat subjective. CARS scores of some of the participants are quite low, when we reviewed the data, we found that these cases did meet the diagnostic criteria of DSM-5. Our clinical diagnosis mainly relies on DSM-5 as the standard. We try to expand the sample size in our research. We prefer to use the semistructured Autism Diagnostic Observation Schedule (ADOS) or the second version of the CARS in further research.

Third, we analyzed many factors but did not include air pollution and many pregnancy-related factors, such as maternal obesity, cesarean section and diabetes. However, a meta-analysis illustrated that pregnancy factors are not as important to the severity of ASD [47]. The air pollution factors need further discussion.

Forth, this is essentially a cross-sectional study. This research does not provide its inherent causal interpretation, thus its predictive ability is likely very limited.

## Conclusion

Many environmental factors affect the severity of ASD. We concluded that non-parental caregivers, low maternal education, gastrointestinal problems and a high serum concentration of lead were predictors that affected the severity of ASD. The objectives of this study were to identify high-risk populations with potentially severe ASD symptoms and to provide increased knowledge and family training guidance for parents with low maternal education levels, which can promote the early diagnosis and treatment of high-risk children and can result in a better prognosis. Achieving such outcomes may require the efforts of the entire autism prevention system at the government level and all medical workers in related fields. Concerns about gastrointestinal symptoms and blood lead levels in children with ASD warrant further research. Future studies should investigate how these factors interact ASD symptoms and help to further uncover the pathogenesis of autism.

## Data Availability

The datasets used and/or analyzed during the current study are available from the corresponding author on reasonable request.

## Abbreviations

ASD: Autism spectrum disorder
CARS: Childhood Autism Rating Scale
DSM-5: Diagnostic and Statistical Manual of Mental Disorders 5th Edition
PAHs: Polycyclic aromatic hydrocarbons
ADHD: Attention deficit hyperactivity disorder
ATEC: Autism Treatment Evaluation Checklist
HPLC: High-performance liquid chromatography
AAS: Atomic absorption spectrometry
SPSS: Statistical Package for the Social Sciences
SDs: Standard deviations
ADOS: Autism Diagnostic Observation Schedule
